# Distal Radius Plates With Coracoid Suture Anchors for the Fixation of Displaced Lateral End Clavicle Fractures

**DOI:** 10.7759/cureus.50720

**Published:** 2023-12-18

**Authors:** Mohammed Ali, Dafalla Elamin, Ahmed Ibrahim, Syed S Mannan

**Affiliations:** 1 Trauma and Orthopaedics, Health Education North East, Newcastle, GBR; 2 Trauma and Orthopaedics, Darlington Memorial Hospital, Darlington, GBR; 3 Trauma and Orthopaedics, North Cumbria University Hospitals NHS Trust, Carlisle, GBR; 4 Orthopaedics and Traumatology, North Cumbria University Hospitals NHS Trust, Carlisle, GBR

**Keywords:** distal radius plate, clavicle fixation, orthopaedics, lateral end clavicle fracture, trauma

## Abstract

Introduction

Management of displaced fractures of the lateral end clavicle has always challenged orthopaedic surgeons, due to a high non-union rate, and difficulty achieving stable fixation allowing early mobilisation. Different methods of fixation have been utilised to provide stability and improve healing and functional outcomes. In this series, we evaluate the results of internal fixation using dorsal distal radius locking plates and coracoid suture anchors.

Patients and methods

We retrospectively reviewed the clinical notes and radiographs of eight consecutive patients with fractures of the lateral end of the clavicle presented to our hospital between January 2016 and December 2017. Patients were treated by open reduction and internal fixation using dorsal distal radius locking plates and coracoid suture anchors.

Results

All patients achieved full range of motion of the shoulder at eight weeks postoperatively. There were no intra-operative complications. Evidence of bone healing was noted in all cases within eight weeks post-operatively. There were no cases of wound complications, metal work irritation or fixation failures. The pre-morbid level of function was restored following the rehab protocol and physiotherapy.

Conclusion

Dorsal distal radius plates with suture anchor fixation appear to be a valuable alternative for the treatment of fractures of the lateral end of the clavicle. Coracoclavicular fixation provided and maintained the reduction of the fracture. Good clinical results can be achieved with a low risk of complications and without the need for metalwork removal.

## Introduction

There are five types of distal end clavicle fractures according to Neer’s classification [1]. Type II and V fractures are considered highly unstable and have many controversies in their management. Type II is sub-categorized into two types: type IIA, in which the fracture occurs medial to the coracoclavicular ligament, and type IIB, in which the fracture occurs more laterally with the coracoclavicular ligament disrupted from the proximal fragment [1]. Type V is similar to type II, which involves an avulsion leaving behind an inferior cortical fragment attached to the coracoclavicular ligament [2]. Initially, Neer suggested using trans-acromial Kirschner wire to stabilise the fracture; however, over the years, several different surgical techniques have been developed for the fixation of the lateral end clavicle fractures [3]. A recent review by Sambandam et al. [2] discussed different fixation methods. These include pre-contoured locking plates [4], hook plates [5], distal radius plates [3], coracoclavicular screws [6], flexible coracoclavicular fixation [7] and intramedullary fixation [8]. However, these techniques were also accompanied by multiple disadvantages, including wire migration, failure of fixation, irritation of soft tissues, including the rotator cuff, infection, fractures and osteoarthritic changes [2]. To provide a good anatomical reduction and maintain stability, we used distal radius locking plates alongside coracoid suture anchor fixation. In this paper, we discuss the technique and the outcomes of surgery in eight patients presented with lateral end clavicle fractures.

## Materials and methods

The retrospective analysis involved a comprehensive review of clinical records and radiographs of patients who presented with lateral end clavicle fractures, managed operatively with distal radius locking plates and coracoid suture anchors. The study included patients receiving this fixation technique while excluding cases managed with alternative fixation methods. Parameters examined in this study included, but not limited to, surgical duration, peri-operative complications, union time, and functional recovery.

**Figure 1:**
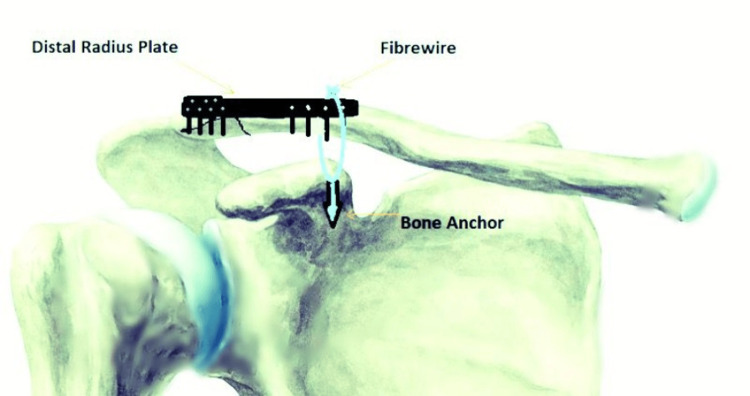
Placement of the bone anchor, distal radius plate and Fibrewire in relation to the lateral end clavicle fracture.

Procedures were conducted with patients in a deck chair under position under general anaesthesia. Preceding the incision, 10 ml of chirocaine with 1:200,000 adrenaline was injected along the lateral clavicle axis following skin marking. Surgical steps involved exposing fracture ends, retracting, elevating the anterior deltoid flap and revealing the coracoid base. A 3.5-mm fibre wire anchor, carrying double-loaded Fiberwire sutures, was inserted into the coracoid base, positioning one limb of each suture behind the clavicle and the other anteriorly. Adjustments included reduction of the fracture, utilising Kirschner wires when necessary. Following reduction, a distal radius locking plate was affixed (Figure 1). This plate was specifically designed to require no additional bending after achieving anatomical reduction. Its minimal profile facilitated soft tissue coverage, a crucial factor in the dorsum of the distal radius, where tendon gliding over the plate is necessary. Subsequent to reduction, previously placed sutures were individually tied over the plate. The wound closure involved layered suturing, and the operated arm was immobilised in a broad sling. Patients were encouraged to engage in gentle, assisted pendulum shoulder movements for four weeks, transitioning to active movements afterward.

Post-operatively, patients underwent a wound assessment at two weeks, which included suture/clip removal, followed by subsequent visits at four and eight weeks for check-ups and radiographic evaluations. This meticulous process aimed to evaluate both the clinical efficacy and the patient's recovery trajectory following the specific fixation method employed for lateral end clavicle fractures.

## Results

**Figure 2:**
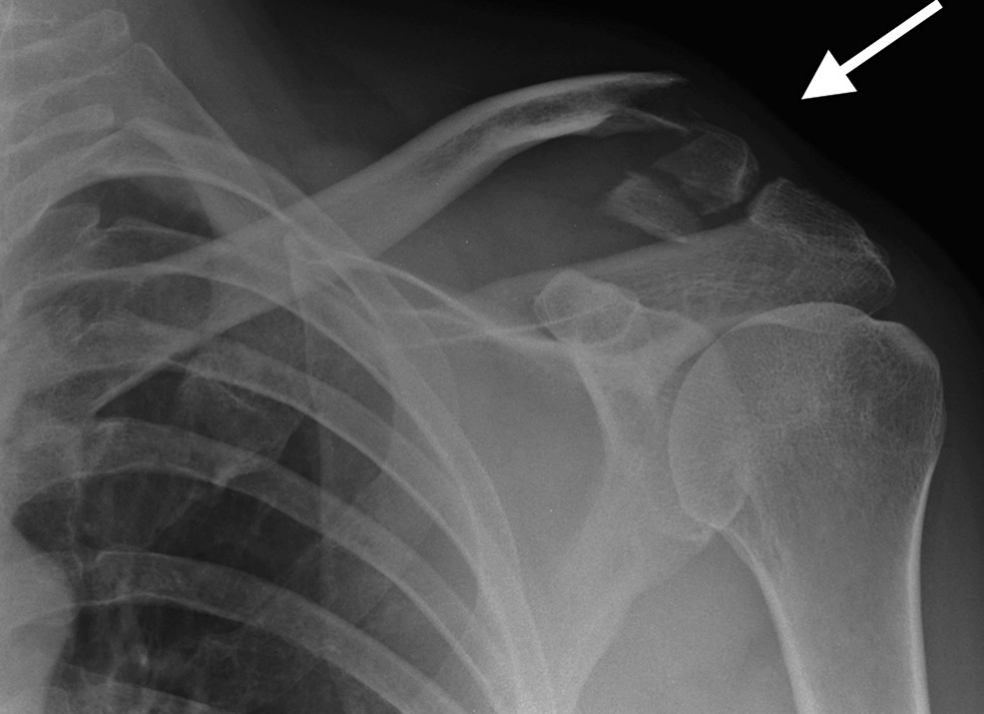
Neer type V fracture.

Eight patients with unstable lateral end clavicle fractures presented to our hospital between January 2016 and December 2017. All injuries were closed with no distal neurovascular deficit. Patients underwent open reduction and internal fixation with distal radius locking plates (Variax, manufactured by Stryker), which allow using seven distal locking screws. In addition, coracoid suture anchors (3.5 mm twin-fix manufactured by Smith and Nephew) were used to fix the medial fragment. Seven cases were Neer type IIA, and one case was type V (Figure 2).

**Figure 3:**
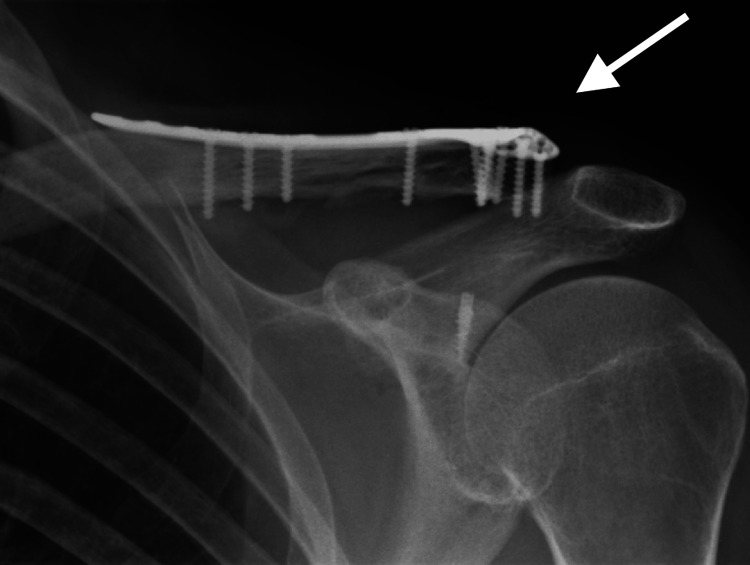
Radiographs at eight weeks.

This series included three males and five females with a mean age of 48 years (range 20 to 77). All fractures occurred due to a fall on the arm. One patient had an associated olecranon fracture, two had facial injuries and the rest were isolated. The mean interval between trauma and surgical treatment was 4.3 days (range: one to five days). The mean surgical time was 62 mins (range 45 to 77 mins). The mean follow-up was six months. Union was achieved in all fractures. At eight weeks, all patients had an evident clinical union (painless, full range of movement (ROM) and improved shoulder strength) alongside callus formation on radiographs (Figure 3).

No further imaging was required in all patients. All patients had a functional range of movement at eight weeks. All patients had returned to normal daily activities and to work without limitations at eight weeks. None of those patients had a failure of fixation, loss of reduction, or wound complication. None had rotator cuff symptoms, and there were no cases of superficial or deep infections.

## Discussion

Lateral end clavicle fractures with disruption of the attachment of the coracoclavicular ligament to the medial clavicular fragment (Neer type II) are considered highly unstable injuries, and the healing outcomes without fixation are generally very poor [1,2]. The fracture heals only when the distracting forces, at both of the fracture ends, are neutralised. Surgeons over the years have described many techniques and methods for achieving reduction and fixation. All the previously described techniques have known drawbacks and complications due to the complexity of the fracture [2]. Currently, locking clavicle plates have been specifically designed for the fixation of these fractures; however, these are more robust in thickness, and as the distal fragment is usually small, soft and comminuted, it is challenging to achieve a satisfactory fixation with fewer locking holes and comminuted softer bone. Add to that, locking clavicle plates can be prominent and compromise the soft tissues [4,9,10]. K-wires with cerclage have been reported to have a high rate of infection and non-union [11], and recently, the clavicle hook plate has been widely abandoned as it is associated with rotator cuff lesions, subacromial impingement and painful subacromial osteolysis, thus will require removal at some point [12].

Volar distal radius locking plates were previously used by Kalamaras et al. [13], Daglar et al. [14], and Herrmann et al. [15]. Although they reported a small series, they demonstrated that it was possible to sufficiently fix this difficult fracture due to new developments in the plate-screw interface. The most significant complication described by these studies was the pull-out of the plate when the plate had too little grip on a small or osteoporotic lateral fragment. In our opinion, pull-out occurred in these cases for these plates because they did not neutralise the downward forces acting on the lateral end of the fracture sufficiently. We found that when supplemented with a coracoid suture anchor fixation, this adds a ‘belt and braces’ fixation in a very simple manner and neutralises the downward forces. The suture anchor is easily applied to the superior central surface of the coracoid by using two bone levers to mark either margin. This technique is much easier than passing a suture or a tape under the coracoid. Furthermore, the suture is very low-profile when knotted around the clavicle and tied over the plate. The distal radius locking plates usually lend themselves to a very good fit without contouring after anatomical reduction. There were no instances of plate pullout observed in the follow-up period of this study. 

The limitations of this study included its retrospective nature; it is a small series, and the treatment was carried out by one consultant. This limited sample size is typically adequate for initial investigations or pilot studies, focusing on assessing feasibility and initial results. However, this constrains the ability to broadly apply the findings, highlighting the need for more extensive studies to reach conclusive results. We also note that the suture anchors around the coracoid can be technically challenging and needs specialised training. 

## Conclusions

In this retrospective study, we achieved excellent initial results with dorsal distal radius locking plates and coracoid suture anchors, although the number of patients included in the study was small. This technique, in our opinion, does not compromise the soft tissues, is easily done and reproducible, and fixes the fracture sufficiently to provide a rigid and stable construct. It enables the initiation of early active motion and does not require a second operation for implant removal.
